# Update on therapeutic strategy for esophageal anastomotic leak: A systematic literature review

**DOI:** 10.1111/1759-7714.14734

**Published:** 2022-12-16

**Authors:** Feng Hua, Dongfeng Sun, Xiaoming Zhao, Xuemin Song, Wenfeng Yang

**Affiliations:** ^1^ Department of Thoracic Surgery Shandong Cancer Hospital and Institute Shandong First Medical University and Shandong Academy of Medical Sciences Jinan China

**Keywords:** anastomotic leakage, esophageal carcinoma, esophagectomy, esophagus, fistula

## Abstract

Anastomotic leak is still a severe complication in esophageal surgery due to high mortality. This article reviews the updates on the treatment of anastomotic leak after esophagectomy in order to provide reference for clinical treatment and research. The relevant studies published in the Chinese Zhiwang, Wanfang, and MEDLINE databases to December 21, 2021 were retrieved, and esophageal carcinoma, esophagectomy, anastomotic leakage, and fistula selected as the keywords. A total of 78 studies were finally included. The treatments include traditional surgical drainage, new reverse drainage trans‐fistula, stent plugging, endoscopic clamping, biological protein glue injection plugging, endoluminal vacuum therapy (EVT), and reoperation, etc. Early diagnosis, accurate classification and optimal treatment can promote the rapid healing of anastomotic leaks. EVT may be the most valuable approach, simultaneously with good commercial prospects. Reoperation should be considered in patients with complex fistula in which conservative treatment is insufficient or has failed.

## Graphical abstract

The aim of this review article is to discuss esophageal anastomotic leak, and describe modern treatment approaches. Endoluminal vacuum therapy (EVT, E‐VAT)may be the most promising therapeutic strategy for the most dreaded complication. EVT (E‐VAT) also has good commercial prospects.

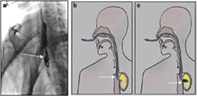

## INTRODUCTION

Esophageal cancer is one of the most common malignant tumors in China.^1^ The key step and mainstay in treating patients is still surgical removal of the esophagus and reconstruction of the upper digestive tract. More and more patients with esophageal cancer receive surgery‐based treatment to achieve long‐term survival.^2^ Since its introduction by Czerny in the 1870s, esophagectomy has been feared for anastomotic leak, which is still the most prominent and dreaded complication and challenge for surgeons up to now. The unremitting exploration for the treatment of anastomotic leak promotes the continuous progress and renewal of the therapeutic concept and applied technology. Here, we review the related updates on the treatment of anastomotic leak in recent years. Our objective is to discuss the most dreaded complication and describe modern treatment approaches to overcome it.

## COMPLEX CLINICAL PRESENTATIONS AND POSSIBLE CAUSES OF ANASTOMOTIC LEAKS

**FIGURE 1:**
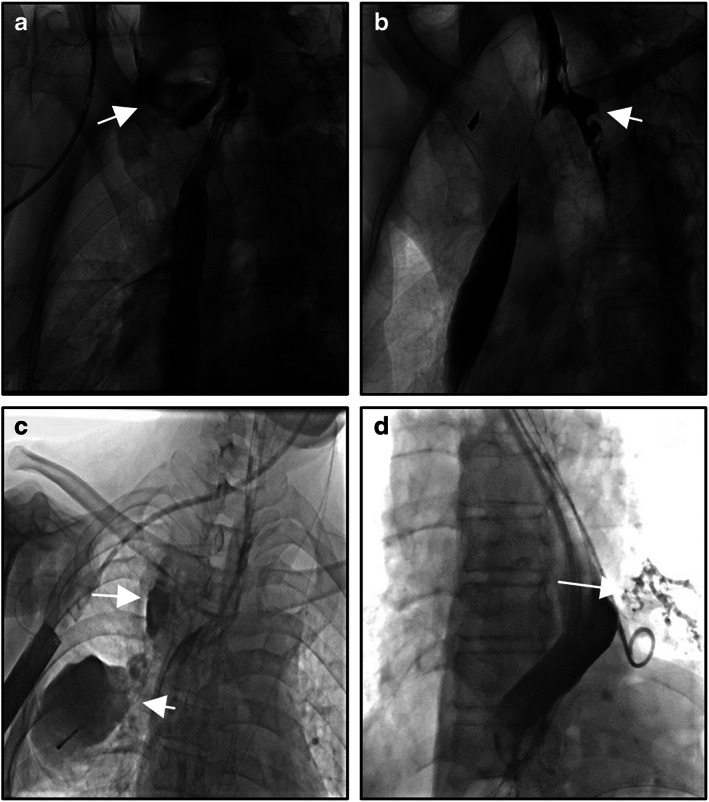
Complex clinical presentation of anastomotic leakage after esophagectomy. (a) The most common and simple fistula, neck cutaneous fistula. (b) Mediastinal fistula. (c) Multiventricular purulent cavity, complex fistula. (d) Broncho‐gastric fistula. The arrow Mark means the leak.

The local defect of esophageal gastric (or jejunum, colon) anastomotic line (or upper, or lower tissues) will lead to destruction of the integrity of digestive lumen, and then enteric contents and saliva leak to the outside of the lumen, which will cause a series of clinical syndromes. Anastomotic leaks may be divided into cervical or thoracic (mediastinal) anastomotic leak according to the location of occurrence (Figure 1a,b). According to the time of occurrence, it can be divided into early, medium and later anastomotic leak. According to the clinical manifestations, it can be divided into simple anastomotic leak and complex anastomotic leak (including multiventricular purulent cavity, tracheal fistula, bronchial‐pulmonary fistula, cardiac or macrovascular fistula, etc.) (Figure 1c,d). According to the presence or absence of positive performance, it can be divided into obvious or concealed anastomotic leak. The clinical manifestation of gastric staple line leaks is consistent with that of anastomotic leaks and is generally included in the category of anastomotic leaks, as the incidence is significantly lower. The high incidence of cervical leak and the high mortality rate of thoracic (mediastinal) leak have always been a major problem in esophageal surgery. With the popularity of minimally invasive technique (MIE) and the cognition of the scope of lymph field dissection, subtotal esophagectomy and cervical anastomosis have become the main surgical criteria. The research on anastomotic leak is more and more focused on the prevention and treatment of cervical anastomotic leak.

Only by fully understanding the risk factors leading to anastomotic leak can we minimize its occurrence. To comprehensively analyze the risk factors of anastomotic leak, first of all, it is emphasized that a precise and scrupulous surgical technique plays a decisive role in the healing of anastomotic tissue. Precise surgical operation needs to ensure firm and stable tissue closure, good blood supply to the stomach or other organs replacing the esophagus and targeted treatment of anastomotic tension.^3^, ^4^, ^5^, ^6^, ^7^, ^8^, ^9^, ^10^, ^11^, ^12^, ^13^, ^14^, ^15^, ^16^, ^17^, ^18^, ^19^, ^20^, ^21^, ^22^, ^23^, ^24^, ^25^, ^26^, ^27^, ^28^, ^29^, ^30^, ^31^, ^32^, ^33^, ^34^, ^35^, ^36^, ^37^, ^38^, ^39^, ^40^, ^41^, ^42^, ^43^, ^44^, ^45^, ^46^, ^47^, ^48^, ^49^, ^50^, ^51^, ^52^, ^53^, ^54^, ^55^, ^56^, ^57^, ^58^, ^59^, ^60^, ^61^, ^62^, ^63^, ^64^, ^65^, ^66^, ^67^, ^68^, ^69^, ^70^, ^71^, ^72^, ^73^, ^74^, ^75^, ^76^, ^77^, ^78^ At present, gastric conduit, especially the thin conduit replacing the esophagus, has been accepted by more and more surgeons. In addition to a higher quality of life, the application of a thin gastric conduit replacing the esophagus also effectively reduces the occurrence of anastomotic leak.^8^ The anastomotic technology with the gastric conduit has also been continuously improved by surgeons. Liu et al. compared the leak rate of end‐to‐end and end‐to‐side esophagogastric anastomosis and found that the end‐to‐end anastomosis further reduced the incidence of anastomotic leaks.^9^

In addition to surgical technical factors, the ability of the patient's own tissue healing, including age, nutritional status, diabetes or other basic disease factors, intra‐ and postoperative hypoperfusion and hypoxia are also potential factors leading to anastomotic leak. Some scholars have reviewed and analyzed the risk factors of anastomotic leaks in the McKeown procedure. The results showed that ASA grade, peripheral vascular disease and renal insufficiency were closely related to cervical anastomotic leaks, and calcification of descending aorta and celiac artery were also independent risk factors.^10^

Among the causes of anastomotic leak, iatrogenic factors such as metal anastomotic nails and hemostatic clips must be emphasized because this mechanical friction may often lead to serious complex fistula, such as macrovascular and tracheobronchial fistula.^11^, ^12^ The possibility of a postoperative gastric ulcer should not be neglected, although its incidence rate is very low.^13^ Maruyama et al. reported a case of esophagotracheal fistula secondary to gastric acid reflux 9 years after esophageal cancer resection. The patient was successfully treated by transposition of the pedicled pectoralis major myocutaneous flap.^14^

## TREATMENT OF ANASTOMOTIC LEAK

Once the anastomotic leak occurs, the contents of the digestive tract extravasate to the tissue space, chest cavity or mediastinum through the defect. The local infection produces a large number of necrotic substances, inflammatory factors, and then causes a series of clinical manifestations by secondary erosion or destruction of important tissues and organs. The continuous accumulation of purulent and corrosive liquid around the defect also cause the continuous nonunion of the defect, thus forming a vicious circle. Aiming at the complicated clinical manifestations and common pathological mechanism, based on the progress of modern medical technology such as medical bioengineering, surgical technology, endoscopy and intervention medicine, the therapeutic method of anastomotic leak is also progressing. To summarize and analyze the current clinical treatment methods, in addition to the different therapeutic ideas for the local anastomotic defect, effective drainage and source control is the basis or mainstay for treating anastomotic leaks.

## SURGICAL DRAINAGE: THE TRADITIONAL TREATMENT

Since surgery was applied for the treatment of esophageal cancer, the basic method used in anastomotic leak for a long time is simply surgical drainage, that is, the so‐called “traditional support and gastrointestinal decompression to reduce reflux, conservative treatment.” For the cervical anastomotic leak, the cervical incision was opened for natural drainage after removing the suture. Most patients can be cured by natural drainage through the incision. Some scholars compared the drainage effect after the neck incision was half‐ or completely‐open, and the results showed that the effect of half‐open drainage after removing part of the suture was better.^15^ For a thoracic (mediastinal) anastomotic leak, percutaneous thoracic drainage is used, and some patients need to undergo complete puncture and catheterization under CT guidance. The method of percutaneous thoracic drainage tube combined with nutrition tube and gastrointestinal decompression tube, referred to as the “traditional three tube method” or “three tubes and one prohibition,” is the basic method of traditional conservative treatment for thoracic anastomotic leak. According to the idea of surgical drainage for anastomotic leak, many scholars have proposed a series of novel drainage methods, such as T tube drainage,^16^ double cannula drainage,^17^ etc. It is emphasized that in conservative treatment, we should be highly vigilant about the timely and effective control of symptoms such as vomiting and cough that increase the pressure in the esophagus.

However, in some cases, cervical anastomotic leak can lead to the posterior mediastinal sinus purulent cavity or multicompartment purulent cavity. Due to obstruction of the thoracic vertebrae or scapula, percutaneous drainage cannot be performed, which is also the main reason that some patients eventually die. The constant consideration of this dilemma has promoted the discovery of the trans‐fistula drainage technique and has profoundly changed the embarrassment of esophageal anastomotic leak treatment.

## TRANS‐FISTULA, A NOVEL DRAINAGE THROUGH ANASTOMTIC DEFECT

**FIGURE 2:**
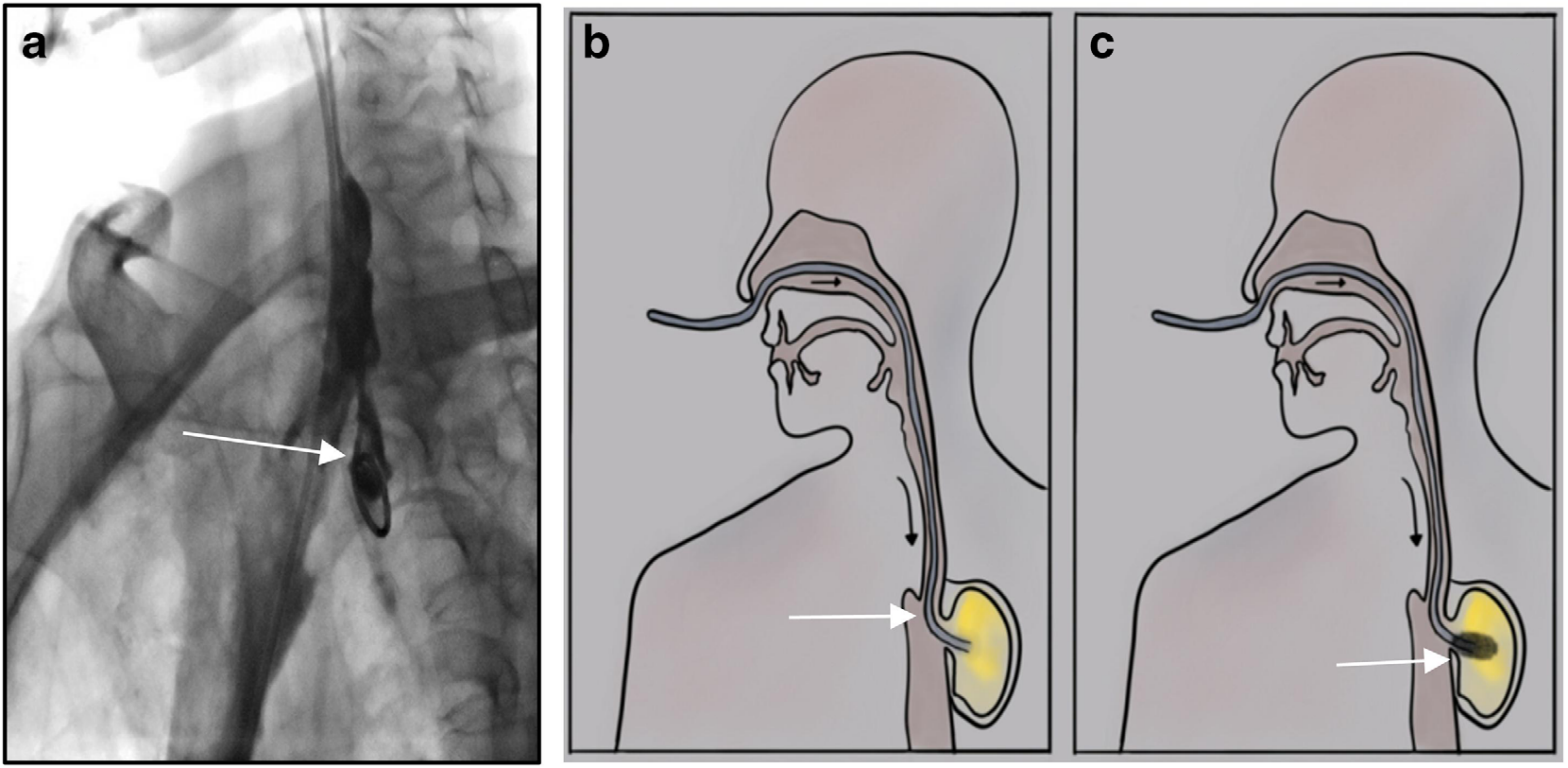
(a) Trans‐fistula drainage involves placing a nasogastric tube through one side of the nostril crossing the leak to implement negative pressure drainage. (b) The pattern diagram of trans‐fistula drainage. (c) Endoluminal vacuum therapy (EVT, E‐VAT), the adjusted sponge is equipped at the distal end of nasogastric tube. The arrow Mark means the leak.

Jiang Ming et al.^18^ in China first reported this new drainage technology in 2006. The therapeutic method is to place a nasogastric tube through one side of the nostril across the defect to implement negative pressure drainage. Four patients with thoracic anastomotic leak were treated and all achieved rapid healing. This technique has been accepted by most esophageal cancer surgeons in China, especially in esophageal cancer surgical centers. At present, most hospitals adopt the method of interventional catheterization, and some hospitals adopt the method under gastroscopy. The nasogastric, nasal nutrition and other tubes are used as the drainage tubes across the defect, and very good treatment results have been achieved.^19^, ^20^, ^21^ The trans‐fistula reverse drainage, enteral nutrition tube and gastrointestinal decompression tube together make up the “the new three tube technology”^21^ (Figure 2a,b).

Interventional or gastroscopic reverse drainage across the defect effectively complements the treatment for these patients be limited to traditional surgical drainage, which is a milestone in the progress of anastomotic leak. However, whether traditional drainage or trans‐fistula reverse drainage, it is mainly suitable for simple anastomotic leak. For patients with complicated anastomotic leak, such as anastomotic (gastric conduit) tracheal fistula or anastomotic (gastric conduit) large vessel fistula, a simple drainage strategy cannot achieve good treatment results.

## ESOPHAGEAL STENTS

The principle of self expandable metallic stent (SEMs) in the treatment of gastrointestinal fistula is to use the released stent to support the tube wall and to block the defect, so as to achieve physical isolation and promote the healing of the leak. With the popularization of SEMs in clinical practice, more and more successful cases have been reported, but many stent‐related complications have also been reported, the most common of which are stent‐related bleeding, stent displacement, and lateral abscess of fistula, etc.^22^, ^23^, ^24^ Some scholars have reviewed the treatment experience of 15 patients with tracheal fistula and found that five were secondary to stent. Therefore, stent occlusion is not recommended for patients with simple anastomotic leak.^25^ Especially, the residual cavity outside the defect after stent implantation may lead to fatal massive bleeding.^26^, ^27^ Some scholars have tried to solve the problem of lateral abscess by placing SEMs and interventional trans‐fistula reverse drainage in the paraesophageal space.^28^ In addition, pharyngeal and laryngeal dysfunction may be induced in patients with cervical anastomotic leak due to the high position of the upper pole after stent implantation, and fatal pulmonary infection may even be caused. At present, esophageal stents are mostly used in patients with esophagotracheal fistula without abscess. Such patients have no thoracic mediastinal infection, and the clinical symptoms can be significantly and quickly relieved after stenting.

Self expandable metallic stents are limited in their clinical application because of many complications and the possibility that they may need to be removed again. Based on the purpose of plugging the defect to achieve physical isolation, a biodegradable stent made of absorbable and degradable new biomaterials can support the lumen for a short time, and will eventually degrade in vivo without having to remove it again. It has the potential to replace the metal stent in order to optimize the application of intracavitary plugging in the treatment of anastomotic leak.^29^ However, there have been few clinical reports on biodegradable esophageal stents, which deserves attention and active research. Similarly, based on the purpose of plugging the defect to achieve physical isolation, Young et al. reported the successful experience of using an umbrella occluder to treat a patient with tracheoesopheageal fistula.^30^

## REOPERATION, DESPITE ITS HIGH MORTALITY AND RISK, IS SOMETIMES THE LAST SALVAGE TREATMENT

Considering the complicated conditions and high risks, most scholars do not choose reoperation for the patients with anastomotic leaks. However, some scholars insist on the applicability, in some special cases, such as a large defect caused by massive gastric tissue necrosis which is difficult to heal in a short time or serious clinical symptoms, unless patients cannot tolerate the second operation. The operation methods mainly include simply defect repair, anastomotic reconstruction and esophageal exclusion.^31^, ^32^ With the application of new technologies such as interventional therapy, endoscopy and negative pressure therapy in the treatment of anastomotic leak, conservative treatment of a “minimally invasive” nature has more prominent advantages than the “trauma” of reoperation. In fact, at present, a nonsurgical treatment strategy is generally adopted in patients with simple anastomotic leak, and reoperation is usually only suitable for patients with complex and serious cases.

Compared with simple cases, complex and severe anastomotic leak is rarely seen in clinic. Once it occurs, the patient is often in a severe, or even critical, condition. The most common are anastomotic (gastric conduit) tracheal fistula and anastomotic (gastric conduit) thoracic aortic fistula, which are also the two most common reasons for patients to die.^33^ There are other rare complex fistulas, such as gastric stump ventricular fistula,^34^ gastric stump pericardial fistula,^35^, ^36^ esophageal spinal cord fistula,^37^ and suppurative spondylitis,^38^ etc.

The secondary trachea or lung fistula caused by anastomotic leak after esophageal cancer surgery has serious symptoms, most of which are accompanied by respiratory failure. Yasuda et al. summarized and analyzed 10 cases of postoperative tracheal fistula, including five cases of tracheal fistula caused by anastomotic leak, located in the cervical or upper thoracic trachea. Four cases of gastric conduit necrosis were located around the carina. One patient with tracheal fistula caused by gastric ulcer had a long‐term fistula. Four cases were cured by operation, three were cured by conservative treatment, and three patients died in hospital.^39^ For the treatment of such patients, if there is no obvious residual cavity between the digestive tract and airway, esophageal or tracheal stent occlusion can quickly alleviate the symptoms. Some scholars have attempted to use trans‐fistula reverse drainage, otherwise high technical requirements are required. For reoperation, living tissue is usually implanted between the esophagus (or gastric conduit) and the airway for surgical repair of the digestive tract and airway. At present, most scholars use pedicled latissimus dorsi flap, intercostal muscle flap and sternocleidomastoid muscle flap for tissue repair.^40^, ^41^, ^42^, ^43^ Other scholars report cases of treating gastric trachea fistula with pedicled pericardial tablets,^44^ and with other biological patches.^45^ A meta‐analysis compared the efficacy of surgery and tracheal stent in the treatment of gastric tracheal fistula. The results showed that the patients in the operation group had a significant survival advantage.^46^ Another scholar reported a successful case of gastric tracheal fistula cured by conservative treatment.^47^ Wang et al. reviewed the literature on anastomotic trachea fistula and reported that using living soft tissue to protect the airway and mediastinal drainage is the key step in surgical treatment. Airway stenting can be used as a temporary solution. In some cases, conservative treatment can also be successful.^48^

Anastomotic thoracic aortic fistula is a very serious complication. Once it occurs, if it cannot be treated quickly, the patient usually dies of hemorrhagic shock in a very short time.^49^ Surgery is the only possible treatment for the rupture of large blood vessels caused by infection and corrosion. It has been reported that endovascular stent isolation therapy has been used for thoracic aorta,^50^ but it is necessary to be vigilant that the bleeding is only temporarily controlled. If the local infection and corrosion caused by the fistula cannot be eliminated, the patient will be later prone to a wider range of large vessel corrosion. Kawamoto et al. summarized the treatment experience of 10 patients with esophagothoracic aortic fistula. They used intraluminal obstruction of thoracic aortic stent to control bleeding, and patients underwent esophagectomy and reconstruction in stage 2 and thoracic aortic replacement in stage 3. It should be noted that the patients included were not anastomotic leak patients after esophageal cancer surgery. However, the treatment strategy adopted in this group provides guidance for the implementation of salvage surgery in patients with fatal massive hemorrhage caused by anastomotic leak.^51^

## NEGATIVE PRESSURE AND ENDOLUMINAL VACUUM THERAPY ( **EVT** , **E‐VAT** )

“Negative pressure” as an independent treatment technique rather than a simple drainage tool was first proposed by scholars of the former Soviet Union in the 1970s. As a formal clinical treatment concept, Wim Fleischmann, from the trauma surgery hospital of Ulm University, Germany, put forward vacuum sealing drainage technology in 1993,^52^, ^53^ and formally completed the evolution of negative pressure from drainage tool to the treatment method itself. In 1997, American surgeon Argenta deeply studied the mechanism of negative pressure wound treatment and put forward the concept of vacuum assisted closure (VAC). At present, the treatment concept of negative pressure wound treatment (NPWT) is mainly used. NPWT was quickly introduced into the treatment of a variety of clinical chronic refractory wounds after it entered the clinic, especially in a variety of diseases that could not be cured in the past.^52^ Relevant studies have also proved the pathological and molecular mechanism of negative pressure technology in rapidly promoting wound healing, mainly involving inducing perfusion changes, micro deformation, bacterial inhibition, angiogenesis, and rapidly promoting the growth of fibrous granulation tissue.^54^, ^55^, ^56^ At present, negative pressure therapy has become the most effective technical choice for wound treatment technology, which has acquired the consensus of international experts and manufacturers' guidelines, and has produced huge related commercial values.^57^

Anastomotic leak is essentially a special kind of wound. According to the anatomical characteristics of digestive tract lumen, the drainage tube at the distal end of which adjusted sponge is equipped, is placed by the endoscope to the defect, and continuous suction is made in the necrotic anastomotic cavity (Figure 2c). It is called endoscopic vacuum assisted closure (e‐vac) or endoluminal vacuum therapy (EVT, E‐VAT), or endoscopic negative pressure therapy (ENPT). It is the latest technology used in gastrointestinal fistula. According to relevant clinical reports, good therapeutic effects have been achieved in both esophageal gastric anastomotic leak and gastric staple line leak.^58^, ^59^, ^60^, ^61^, ^62^ Laukoetter et al. used this technique to prospectively study the treatment of 52 patients. A total of 52 patients, of which 37 were male and 15 were female, aged 41–94 years, had anastomotic leak secondary to esophagectomy or gastrectomy, iatrogenic esophageal perforation, and Boerhaave syndrome. After diagnosis, the polyurethane sponge was positioned by the endoscope, 390 interventions were conducted and a continuous negative pressure of 125 mmHg was applied to the EVT (ENPT) system. The sponge was replaced twice a week. The results showed that the average sponge replacement frequency was six times, the average treatment time was 22 days, and 94.2% of the patients recovered. EVT failed in three patients (6%). Two of these patients died of EVT related bleeding. Four stenoses were observed during up to 4 years of follow‐up.^63^ Rausa et al. compared the results of 163 patients with esophageal anastomotic leak and perforation treated with EVT (ENPT) or SEMS, respectively. The results showed that EVT was significantly better than SEMs in defect closure rate, treatment time, complication rate and clinical mortality. The authors believe that EVT may be the first choice for esophageal anastomotic leak and perforation.^64^ Valli et al. combined the advantages of EVT (ENPT) and SEMS, tried sponge stents, and treated 12 patients with upper gastrointestinal fistula. Seven patients received first‐line treatment, with a success rate of 71.4% (5/7), and five patients had second‐line treatment, with a success rate of 80% (4/5).^65^ Scott et al. conducted a comparative study on the pig model. Compared with the control pigs, the experimental pigs treated with intraluminal vacuum were killed on the seventh day. It was observed that the defect had healed, showing outstanding therapeutic value.^66^ Virgilio et al. reviewed 29 research reports on EVT (ENPT) in the treatment of upper gastrointestinal anastomotic leak in 2018, comprising 209 patients with 17 retrospective studies, six prospective studies and six case reports. The conclusion is clear and definite. EVT is a very useful method for the treatment of postoperative anastomotic leak in EC/GC patients. Almost all types of anastomotic leak (asymptomatic or symptomatic, small or large defect) appear to be suitable for this technique.^67^ Two years later, the meta‐analysis of Scognamiglio et al. reached the same conclusion.^68^ The follow‐up gastrointestinal quality of life index score showed that patients treated with ENPT (EVT) had higher long‐term quality of life.^69^

In addition to the outstanding effect in the treatment of upper gastrointestinal anastomotic leak, ENPT (EVT) has also achieved the effect of promoting rapid healing in the treatment of intestinal and ureteral anastomotic leak.^70^, ^71^, ^72^ This shows that ENPT (EVT) has a wide range of indications and a reliable therapeutic effect.

With regard to the concept of “negative pressure treatment,” it is necessary to focus on the application of materials to maintain negative pressure. Since Fleischmann applied polymer porous foam materials, polyurethane is currently the main clinical therapeutic material. However, relevant studies have broadened the applicable materials for negative pressure treatment. Now it is clear that no matter what materials or methods are used (other polymer porous foam materials, gauze, or even natural plant loofah collaterals), as long as they can provide stable negative pressure maintenance, they can achieve the same treatment effect.^73^ The special anatomical characteristics of anastomotic leak determine that the more appropriate and effective endoluminal negative pressure treatment tools or systems to replace the current polyurethane foam may be a promising direction to solve the long‐standing obstacle in the future. Huzhendong et al. treated 19 patients with anastomotic leak after esophageal cancer surgery with the method of negative pressure. A nasal purulent drainage tube was externally connected with 125 mmHg continuous negative pressure equipment, and rapid healing was achieved in 19 patients.^74^

## ENDOSCOPIC THERAPY, CLIPPING AND PROTEIN GLUE INJECTION OCCLUSION

Endoscopic clipping therapy refers to the minimally invasive treatment with suture or metal or biological clips to directly close the anastomotic defect under gastroscope. Rodella et al. used titanium clips to treat anastomotic leak after esophagectomy under endoscopy for the first time.^75^ Five of the seven patients were successfully clamped at one time, and the other two patients needed endoscopic treatment two and three times, respectively. All seven patients were cured by endoscopic therapy. However, it should be noted that before titanium clip clamping, the infection should be controlled, edema eliminated, and necrotic tissue should be removed. Some scholars believe that due to the limited opening of titanium clip, this method is only applicable to a defect with a diameter <1 cm.

Medical fibrin glue is a kind of protein glue extracted from animal blood. Its main mechanism is to simulate human blood coagulation. It is often used to stop bleeding on the wound surface and seal defect tissue. For an anastomotic leak, after washing off the pus and coating with normal saline under gastroscopy, biological protein glue can be injected into a small anastomotic leak until the leak is completely filled. On the one hand, it can block and prevent the continuous corrosion of digestive contents. On the other hand, the fibrinogen in the biological protein glue will form reticular fibrin, which not only covers the wound, reduces exudation and infection, but also creates a growth matrix for fibroblasts, which is conducive to the growth of granulation tissue and promotes the healing of defect. For a large defect, it is difficult to heal by one‐time plugging, but it can narrow the cavity. After multiple plugging, the curative effect is good. Nakagawa et al. and other scholars have reported successful cases of sealing a gastric staple line leak or anastomotic leak with biological protein glue.^76^, ^77^ In another study, vicryl mesh containing fibrin glue was injected under gastroscopy, and 13 of the 15 patients were clinically cured.^78^

## CONCLUSION

Surgery is still the main treatment for esophageal cancer in the foreseeable future. With the expansion of operative indication, more and more high‐risk patients will receive esophagectomy. As a serious complication that cannot be completely avoided, anastomotic leak seriously affects the recovery of patients and restricts esophagectomy itself. Timely diagnosis, accurate classification of anastomotic leak, and appropriate technology can easily and quickly overcome the barrier. Endoluminal vacuum therapy (EVT, E‐VAT) may be the most promising method for the treatment of anastomotic leak in the future, which is very worthy of attention and further research. The new negative pressure system and applied materials adapted to anatomical characteristics of digestive tract have good commercial prospects.

## AUTHOR CONTRIBUTIONS

Feng Hua, Wenfeng Yang contributed to the conception of the article and wrote the manuscript. Dongfeng Sun, Xiaoming zhao, Xuemin song helped manuscript preparation and constructive discussions.

## CONFLICT OF INTEREST

The authors declare that there is no conflict of interest.

## References

[1] Zhou J‐c , Zhang R‐s , Zhang S‐w , Chen R, Wang S‐m, Sun K‐x, et al. Analysis on the trend of esophageal cancer incidence and age change in cancer registration areas of China, 2000 to 2015. Chin J Cancer Prev Treat. 2020;27(18):1437–42.

[2] Allemani C , Matsuda T , Di Carlo V , Harewood R, Matz M, Niksik M, et al. Global surveillance of trends in cancer survival 2000–14 (CONCORD‐3): analysis of individual records for 37 513 025 patients diagnosed with one of 18 cancers from 322 population‐based registries in 71 countries. Lancet. 2018;391(10125):1023–75. 29395269 10.1016/S0140-6736(17)33326-3 PMC5879496

[3] Raymond DP . The esophageal anastomosis: traditional methods to prevent leak. J Gastrointest Surg. 2009;13(9):1555–7. 19415397 10.1007/s11605-009-0907-6

[4] Wang S‐j , Wang Q‐z . Carcinoma of the esophagus and gastric cardia. Beijing: People's Health Publishing House; 2008. p. 258–9.

[5] Ikeda Y , Niimi M , Kan S , Shatari T , Takami H , Kodaira S . Clinical significance of tissue blood flow during esophagectomy by laser Doppler flowmetry. J Thorac Cardiovasc Surg. 2001;122(6):1101–6. 11726885 10.1067/mtc.2001.117835

[6] Xiao W‐z , Shi J‐h . Clinical experience of 303 consecutive cases of esophageal carcinoma without anastomotic leakage after operation. Jiangsu Med J. 2018;44(3):345–7.

[7] Huang Q , Fu X‐n , Zu Y‐k , Ma L‐c, Zhang N, Sun W, et al. Clinical significance of intraoperative blood flow measurement of tunica serosa gastria in selecting superior location of gastro‐esophageal anastomosis. J Clin Surg. 2006;10:640–2.

[8] Kuang B‐j , Hu Y , Yu N , Ma L, Song S‐q. Curative effect of thin‐tube transgastric esophagus on prevention of esophagogastric anastomotic leakage and its effect on short‐term quality of life after operation. Hebei Med. 2019;25(4):639–43.

[9] Liu E‐y , He D , Sun W . Application of reverse puncture in end‐to‐end cervical esophago‐gastric anastomosis without nasogastric tube in minimally invasive esophagectomy in patients with esophageal cancer. Tumor. 2015;35(7):801–5.

[10] Zhao G‐f , Zhang K‐p , Gao S‐g . Analysis of the risk factors for postoperative cervical anastomotic leakage after McKeown's esophagectomy. Chin J Oncol. 2017;39(4):287–92. 10.3760/cma.j.issn.0253-3766.2017.04.010 28550670

[11] Chen YY , Chang JM , Lai WW . Tracheo‐neo‐esophageal fistula caused by exposed metallic staples erosion. Ann Thorac Surg. 2012;94(4):1375 author reply 1375‐6. 22487402 10.1016/j.athoracsur.2012.03.089

[12] Han Y , Yang S , Huang W , Wang Z , Li H . A hem‐o‐Lok‐induced tracheoesophageal fistula cured by temporary airway stenting modified with three‐dimensional printing. Ann Thorac Surg. 2018;106(4):e219–21. 29763597 10.1016/j.athoracsur.2018.04.037

[13] McDermott M , Hourihane DO . Fatal non‐malignant ulceration in the gastric tube after oesophagectomy. J Clin Pathol. 1993;46(5):483–5. 8320336 10.1136/jcp.46.5.483 PMC501268

[14] Maruyama K , Motoyama S , Okuyama M , Sato Y , Hayashi K , Minamiya Y , et al. Esophagotracheal fistula caused by gastroesophageal reflux 9 years after esophagectomy. World J Gastroenterol. 2007;13(5):801–3. 17278207 10.3748/wjg.v13.i5.801 PMC4066017

[15] Wu S‐m , Wang M‐w , Chen Z‐y , Yang G‐y. Effect comparison of open and semi‐open drainage for healing of cervical fistula after esophageal cancer surgery. Cancer Res Clin. 2019;06:419–21.

[16] Wang C‐g , Ye Y‐x , Lou X‐b , Lin L, Xu X, et al. Treatment of 9 cases of intrathoracic anastomotic fistula after esophagectomy. Chin J Gastrointes Surg. 2007;04:318.

[17] Yao S , Liu C‐h , Yang N , Li Z‐d, Li D‐m, Dong G‐h, et al. Clinical research of the treatment on cervical esophagus fistula with low negative pressure suction by double caping pipe. Clin Med China. 2016;32(3):251–4.

[18] Jiang M , Yu M‐f , Xu L , Yin G‐w. Application of interventional catheterization in the treatment of anastomotic leakage after esophageal cancer surgery. Hebei Med J. 2006;09:809–10.

[19] Gou G‐z , Li Y‐s , Ren W‐w . The comprehend of treatment through nose‐orificium fistulae flyback chest drainage. Chin Foreign Med Res. 2011;9(3):7–8.

[20] Zhao M , Wu G , Han X‐w . Interventional treatment of 12 cases of esophagogastric anastomotic fistula with "three tube method" through nasal cavity. J Zhengzhou Univ (Med Sci). 2007;06:1193–5.

[21] Yin G‐w , Chen S‐x , Feng C‐w , Zhang Q, Hu z‐d, Xi W, et al. The new "3‐tube" interventional therapy for gastroesophageal anastomotic fistula. J Interv Radiol. 2008;17(11):812–4.

[22] Rodrigues‐Pinto E , Pereira P , Ribeiro A , Moutinho‐Ribeiro P , Lopes S , Macedo G . Self‐expanding metal stents in postoperative esophageal leaks. Rev Esp Enferm Dig. 2016;108(3):133–7. 26786230 10.17235/reed.2016.3987/2015

[23] Plum PS , Herbold T , Berlth F , Christ H , Alakus H , Bludau M , et al. Outcome of self‐expanding metal stents in the treatment of anastomotic leaks after Ivor Lewis esophagectomy. World J Surg. 2019;43(3):862–9. 30377723 10.1007/s00268-018-4832-2

[24] van den Berg MW , Kerbert AC , van Soest EJ , Schwartz MP , Bakker CM , Gilissen LPL , et al. Safety and efficacy of a fully covered large‐diameter self‐expanding metal stent for the treatment of upper gastrointestinal perforations, anastomotic leaks, and fistula. Dis Esophagus. 2016;29(6):572–9. 25893629 10.1111/dote.12363

[25] Palmes D , Kebschull L , Bahde R , Senninger N , Pascher A , Laukötter MG , et al. Management of nonmalignant tracheo‐ and bronchoesophageal fistula after esophagectomy. Thorac Cardiovasc Surg. 2021;69(3):216–22. 32114691 10.1055/s-0039-1700970

[26] Schweigert M , Dubecz A , Stadlhuber RJ , Muschweck H , Stein HJ . Risk of stent‐related aortic erosion after endoscopic stent insertion for intrathoracic anastomotic leaks after esophagectomy. Ann Thorac Surg. 2011;92(2):513–8. 21592460 10.1016/j.athoracsur.2011.02.083

[27] Schweigert M , Solymosi N , Dubecz A , Stadlhuber RJ , Muschweck H , Ofner D , et al. Endoscopic stent insertion for anastomotic leakage following oesophagectomy. Ann R Coll Surg Engl. 2013;95(1):43–7. 23317727 10.1308/003588413X13511609956255 PMC3964637

[28] Schubert D , Kuhn R , Lippert H , Pross M . Selfexpandable metal stent for the treatment of a large anastomotic insufficiency after esophageal resection. Z Gastroenterol. 2003;41(2):181–4. 12592601 10.1055/s-2003-37310

[29] Kochhar R , Samanta J , Basha J , Verma A , Choudhuri G , Lakhtakia S , et al. Biodegradable stents for caustic esophageal strictures: do they work. Dysphagia. 2017;32(4):575–82. 28444489 10.1007/s00455-017-9800-8

[30] Young JA , Shimi SM , Alijani A , Patil PV , Bhat R . Occlusion of a neo‐esophageal‐bronchial fistula using the Amplatzer vascular plug 2. Diagn Interv Radiol. 2013;19(3):259–62. 23302283 10.5152/dir.2013.026

[31] Marty‐Ané CH , Prudhome M , Fabre JM , Domergue J , Balmes M , Mary H . Tracheoesophagogastric anastomosis fistula: a rare complication of esophagectomy. Ann Thorac Surg. 1995;60(3):690–3. 7677505 10.1016/0003-4975(95)00284-r

[32] Buskens CJ , Hulscher JB , Fockens P , Obertop H , van Lanschot JJ . Benign tracheo‐neo‐esophageal fistulas after subtotal esophagectomy. Ann Thorac Surg. 2001;72(1):221–4. 11465183 10.1016/s0003-4975(01)02701-1

[33] Bartels HE , Stein HJ , Siewert JR . Respiratory management and outcome of non‐malignant tracheo‐bronchial fistula following esophagectomy. Dis Esophagus. 1998;11(2):125–9. 9779370 10.1093/dote/11.2.125

[34] Rana ZA , Hosmane VR , Rana NR , Emery DL , Goldenberg EM , Gardner TJ . Gastro‐right ventricular fistula: a deadly complication of a gastric pull‐through. Ann Thorac Surg. 2010;90(1):297–9. 20609805 10.1016/j.athoracsur.2009.11.042

[35] Kim WJ , Choi EJ , Oh YW , Kim KT , Kim CW . Gastropericardial fistula‐induced pyopneumopericardium after esophagectomy with esophagogastrectomy. Ann Thorac Surg. 2011;91(1):e10–1. 21172468 10.1016/j.athoracsur.2010.09.082

[36] Park S , Kim JH , Lee YC , Chung JB . Gastropericardial fistula as a complication in a refractory gastric ulcer after esophagogastrostomy with gastric pull‐up. Yonsei Med J. 2010;51(2):270–2. 20191021 10.3349/ymj.2010.51.2.270 PMC2824874

[37] Mecklenburg I , Probst A , Messmann H . Esophagospinal fistula with spondylodiscitis and meningitis after esophagectomy with gastric pull‐up. J Gastrointest Surg. 2008;12(2):394–5. 17955314 10.1007/s11605-007-0363-0

[38] Kakuta T , Kosugi S , Kanda T , Hatakeyama K . Purulent spondylitis related to anastomotic fistula after esophageal cancer surgery. Interact Cardiovasc Thorac Surg. 2010;11(2):204–6. 20442207 10.1510/icvts.2010.235515

[39] Yasuda T , Sugimura K , Yamasaki M , Miyata H , Motoori M , Yano M , et al. Ten cases of gastro‐tracheobronchial fistula: a serious complication after esophagectomy and reconstruction using posterior mediastinal gastric tube. Dis Esophagus. 2012;25(8):687–93. 22292530 10.1111/j.1442-2050.2011.01309.x

[40] Narita K , Iwanami H , Ikeda N , Sakonji M , Shinohara Y , Tsuboi E . A successfully treated case of empyema with a large tracheal fistula after a radical operation of esophageal cancer by fixation and plombage with major pectoral muscle flap. Nihon Kyobu Geka Gakkai Zasshi. 1992;40(8):1254–60. 1402170

[41] Nardella JE , Van Raemdonck D , Piessevaux H , Deprez P, Droissart R, Staudt JP, et al. Gastro‐tracheal fistula—unusual and life threatening complication after esophagectomy for cancer: a case report. J Cardiothorac Surg. 2009;4:69. 19943963 10.1186/1749-8090-4-69 PMC2788546

[42] Arimoto J , Hatada A , Kawago M , Nishimura O , Maebeya S , Okamura Y . Closure of esophagotracheal fistula after esophagectomy for esophageal cancer. Gen Thorac Cardiovasc Surg. 2015;63(11):636–9. 26189183 10.1007/s11748-015-0537-8

[43] Bertheuil N , Cusumano C , Meal C , Harnoy Y , Watier E , Meunier B . Skin perforator flap pedicled by intercostal muscle for repair of a tracheobronchoesophageal fistula. Ann Thorac Surg. 2017;103(6):e571–3. 28528074 10.1016/j.athoracsur.2016.12.054

[44] Song SW , Lee HS , Kim MS , Lee JM , Kim JH , Zo JI . Repair of gastrotracheal fistula with a pedicled pericardial flap after Ivor Lewis esophagogastrectomy for esophageal cancer. J Thorac Cardiovasc Surg. 2006;132(3):716–7. 16935147 10.1016/j.jtcvs.2006.05.030

[45] Shi H , Wang WP , Gao Q , Chen LQ . Single‐stage surgical repair of airway gastric fistula after esophagectomy. J Cardiothorac Surg. 2014;9:30. 24506968 10.1186/1749-8090-9-30 PMC3922588

[46] Li Y , Wang Y , Chen J , Li Z , Liu J , Zhou X , et al. Management of thoracogastric airway fistula after esophagectomy for esophageal cancer: a systematic literature review. J Int Med Res. 2020;48(5):300060520926025. 32459126 10.1177/0300060520926025 PMC7278110

[47] Martin‐Smith JD , Larkin JO , O'Connell F , Ravi N , Reynolds JV . Management of gastro‐bronchial fistula complicating a subtotal esophagectomy: a case report. BMC Surg. 2009;9:20. 20030856 10.1186/1471-2482-9-20 PMC2803445

[48] Wang C , Yang X , Zhao J , Chen Q . Postesophagectomy airway‐gastric fistula successfully treated with subcutaneous fascia flap, tracheal reconstruction, and gastric fistula drainage: a case report and literature review. J Cancer Res Ther. 2016;12(Supplement):C225–7. 28230022 10.4103/0973-1482.200599

[49] Molina‐Navarro C , Hosking SW , Hayward SJ , Flowerdew AD . Gastroaortic fistula as an early complication of esophagectomy. Ann Thorac Surg. 2001;72(5):1783–8. 11722100 10.1016/s0003-4975(00)02569-8

[50] Sato O , Miyata T , Matsubara T , Shigematsu H , Yasuhara H , Ishimaru S . Successful surgical treatment of aortogastric fistula after an esophagectomy and subsequent endovascular graft placement: report of a case. Surg Today. 1999;29(5):431–4. 10333413 10.1007/BF02483034

[51] Kawamoto S , Sato M , Motoyoshi N , Kumagai K , Adachi O , Saito T , et al. Outcomes of a staged surgical treatment strategy for aortoesophageal fistula. Gen Thorac Cardiovasc Surg. 2015;63(3):147–52. 25204679 10.1007/s11748-014-0472-0

[52] Fleischmann W , Strecker W , Bombelli M , Kinzl L . Vacuum sealing as treatment of soft tissue damage in open fractures. Unfallchirurg. 1993;96(9):488–92. 8235687

[53] Fleischmann W , Becker U , Bischoff M , Hoekstra H . Vacuum sealing: indication, technique, and results. Eur J Orthop Surg Traumatol. 1995;5(1):37–40. 24193271 10.1007/BF02716212

[54] Orgill DP , Bayer LR . Negative pressure wound therapy: past, present and future. Int Wound J. 2013;10(Suppl 1):15–9. 24251839 10.1111/iwj.12170 PMC7950903

[55] Chen D , Zhao Y , Li Z , Shou K , Zheng X , Li P , et al. Circulating fibrocyte mobilization in negative pressure wound therapy. J Cell Mol Med. 2017;21(8):1513–22. 28211211 10.1111/jcmm.13080 PMC5542905

[56] Ma Z , Li Z , Shou K , Jian C , Li P , Niu Y , et al. Negative pressure wound therapy: regulating blood flow perfusion and microvessel maturation through microvascular pericytes. Int J Mol Med. 2017;40(5):1415–25. 28901392 10.3892/ijmm.2017.3131 PMC5627868

[57] Birke‐Sorensen H , Malmsjo M , Rome P , Hudson D , Krug E , Berg L , et al. Evidence‐based recommendations for negative pressure wound therapy: treatment variables (pressure levels, wound filler and contact layer)—steps towards an international consensus. J Plast Reconstr Aesthet Surg. 2011;64 Suppl:S1–16. 21868296 10.1016/j.bjps.2011.06.001

[58] Leeds SG , Burdick JS . Management of gastric leaks after sleeve gastrectomy with endoluminal vacuum (E‐Vac) therapy. Surg Obes Relat Dis. 2016;12(7):1278–85. 27178614 10.1016/j.soard.2016.01.017

[59] Kuehn F , Schiffmann L , Janisch F , Schwandner F , Alsfasser G , Gock M , et al. Surgical endoscopic vacuum therapy for defects of the upper gastrointestinal tract. J Gastrointest Surg. 2016;20(2):237–43. 26643296 10.1007/s11605-015-3044-4

[60] Smallwood NR , Fleshman JW , Leeds SG , Burdick JS . The use of endoluminal vacuum (E‐Vac) therapy in the management of upper gastrointestinal leaks and perforations. Surg Endosc. 2016;30(6):2473–80. 26423414 10.1007/s00464-015-4501-6

[61] Kuehn F , Loske G , Schiffmann L , Gock M , Klar E . Endoscopic vacuum therapy for various defects of the upper gastrointestinal tract. Surg Endosc. 2017;31(9):3449–58. 28078463 10.1007/s00464-016-5404-x

[62] Bludau M , Fuchs HF , Herbold T , Maus MKH , Alakus H , Popp F , et al. Results of endoscopic vacuum‐assisted closure device for treatment of upper GI leaks. Surg Endosc. 2018;32(4):1906–14. 29218673 10.1007/s00464-017-5883-4

[63] Laukoetter MG , Mennigen R , Neumann PA , Dhayat S , Horst G , Palmes D , et al. Successful closure of defects in the upper gastrointestinal tract by endoscopic vacuum therapy (EVT): a prospective cohort study. Surg Endosc. 2017;31(6):2687–96. 27709328 10.1007/s00464-016-5265-3

[64] Rausa E , Asti E , Aiolfi A , Bianco F , Bonitta G , Bonavina L . Comparison of endoscopic vacuum therapy versus endoscopic stenting for esophageal leaks: systematic review and meta‐analysis. Dis Esophagus. 2018;31(11). 10.1093/dote/doy060 29939229

[65] Valli PV , Mertens JC , Kröger A , Gubler C, Gutschow C, Schneider PM, et al. Stent‐over‐sponge (SOS): a novel technique complementing endosponge therapy for foregut leaks and perforations. Endoscopy. 2018;50(2):148–53. 29186638 10.1055/s-0043-120442

[66] Scott RB , Ritter LA , Shada AL , Feldman SH , Kleiner DE . Endoluminal vacuum therapy for gastrojejunal anastomotic leaks after roux‐en‐Y gastric bypass: a pilot study in a swine model. Surg Endosc. 2016;30(11):5147–52. 26928190 10.1007/s00464-016-4823-z

[67] Virgilio E , Ceci D , Cavallini M . Surgical endoscopic vacuum‐assisted closure therapy (EVAC) in treating anastomotic leakages after major resective surgery of esophageal and gastric cancer. Anticancer Res. 2018;38(10):5581–7. 30275175 10.21873/anticanres.12892

[68] Scognamiglio P , Reeh M , Karstens K , Bellon E , Kantowski M , Schön G , et al. Endoscopic vacuum therapy versus stenting for postoperative esophago‐enteric anastomotic leakage: systematic review and meta‐analysis. Endoscopy. 2020;52(8):632–42. 32316043 10.1055/a-1149-1741

[69] Dhayat SA , Schacht R , Mennigen R , Palmes D , Vogel T , Vowinkel T , et al. Long‐term quality of life assessment after successful endoscopic vacuum therapy of defects in the upper gastrointestinal tract quality of life after EVT. J Gastrointest Surg. 2019;23(2):280–7. 30430432 10.1007/s11605-018-4038-9

[70] Loske G , Müller C . Endoscopic vacuum‐assisted closure of upper intestinal anastomotic leaks. Gastrointest Endosc. 2009;69(3 Pt 1):601–2. author reply 602. 19231507 10.1016/j.gie.2008.06.058

[71] Boulanger K , Lemaire V , Jacquemin D . Vacuum‐assisted closure of enterocutaneous fistula. Acta Chir Belg. 2007;107(6):703–5. 18274191 10.1080/00015458.2007.11680153

[72] Denzinger S , Luebke L , Burger M , Kessler S , Wieland WF , Otto W . Vacuum‐assisted closure therapy in ureteroileal anastomotic leakage after surgical therapy of bladder cancer. World J Surg Oncol. 2007;5:41. 17430598 10.1186/1477-7819-5-41 PMC1855326

[73] Cocjin H , Jingco J , Tumaneng F , Coruña J . Wound‐healing following negative‐pressure wound therapy with use of a locally developed AquaVac system as compared with the vacuum‐assisted closure (VAC) system. J Bone Joint Surg Am. 2019;101(22):1990–8. 31764361 10.2106/JBJS.19.00125

[74] Ma J‐j , Li L , Xu J‐l , Hu Z‐d, Y‐Y, Zhang Q, et al. Application and nursing of negative pressure sealing drainage technique in anastomotic leakage after esophageal cancer operation. Chin J Surg Oncol. 2017;9(5):339–41.

[75] Rodella L , Laterza E , De Manzoni G , Kind R, Lombardo F, Catalano F, et al. Endoscopic clipping of anastomotic leakages in esophagogastric surgery. Endoscopy. 1998;30(5):453–6. 9693892 10.1055/s-2007-1001307

[76] Nakagawa M , Seki M , Koike J , Kanai T . Gastric tube‐to‐pleural fistula seventeen months after esophagectomy: successful endoscopic treatment of an unusual complication. Jpn J Thorac Cardiovasc Surg. 2005;53(10):569–72. 16279590 10.1007/s11748-005-0070-2

[77] Bianchi Cardona A , Hidalgo Grau LA , Feliu Canaleta J , Espin Alvarez F , Suñol SJ . Postoperative cervical anastomotic fistula treated with a biologic glue. Eur J Surg Oncol. 2005;31(10):1222–3. 16226420 10.1016/j.ejso.2005.08.001

[78] Böhm G , Mossdorf A , Klink C , Klinge U , Jansen M , Schumpelick V , et al. Treatment algorithm for postoperative upper gastrointestinal fistulas and leaks using combined vicryl plug and fibrin glue. Endoscopy. 2010;42(7):599–602. 20432210 10.1055/s-0029-1244165

